# Mn_3_O_4_ Nanozyme Soaking Improved Wheat Germination and Yield Under Salt Stress

**DOI:** 10.3390/plants15142124

**Published:** 2026-07-09

**Authors:** Linbo Zhao, Wenrui Qi, Jiahao Liu, Linfeng Bao, Mengyang Li, Mengke Du, Tingyong Mao, Wei Sang, Pengpeng Liu, Jiangbo Li, Yunlong Zhai, Desheng Wang

**Affiliations:** 1 College of Agriculture, Tarim University, Alar 843300, China; zhaolinbo1998@163.com (L.Z.); 19838580805@163.com (W.Q.); 13163247758@163.com (J.L.); blfbzb@163.com (L.B.); 18746229985@163.com (M.L.); d3384140167@163.com (M.D.); tymao@taru.edu.cn (T.M.); 2 Key Laboratory of Tarim Oasis Agriculture, Tarim University, Ministry of Education, Alar 843300, China; 3 Crops Research Institute, Xinjiang Academy of Agricultural and Reclamation Sciences, Shihezi 832000, China; nkysangwei@163.com (W.S.); nkylpp@163.com (P.L.); 18699160581@163.com (J.L.)

**Keywords:** wheat, salinity, Mn_3_O_4_ nanoparticles, yield, ROS, sugar metabolism

## Abstract

Salinity is a major factor limiting the increase in crop yield around the world. Wheat starch and straw are important raw materials for producing bioethanol, and their productivity is adversely affected by salinity. Polyacrylic acid-modified Mn_3_O_4_ nanoparticles (PMO) have been reported to improve crop tolerance to stressors, including salt stress. Therefore, this study aimed to elucidate the mechanism by which seed soaking with PMOs enhances wheat salt tolerance. PMO seed soaking promoted wheat germination (increase of 30.9%) under salt stress, and seedlings treated with PMO seed soaking had a higher fresh weight (increase of 31.4%). PMO seed soaking increased the POD activity (by 31.9%) but decreased superoxide dismutase and catalase activities of wheat seeds and the O_2_^−^ and H_2_O_2_ contents (by 25.0 and 71.4%, respectively). Furthermore, PMO soaking increased the ATP, NADPH, and NADH contents (by 367.6, 212.0, and 283.2%, respectively) by regulating sugar metabolism and enhanced the ascorbic acid–glutathione cycle. Additionally, the PMO soaking treatment optimized energy allocation in wheat under salt stress and increased yield (by 9.7%). PMO modulated sugar metabolism, thereby optimizing energy allocation to plant growth and the antioxidant system, which enhanced wheat germination and yield formation under salt stress.

## 1. Introduction

Salt stress is a critical issue decreasing crop yields [1]. The starch and straw of wheat are important raw materials for producing bioethanol, which is significant for industry development [2,3]. In China, 60% of wheat (*Triticum aestivum* L.) is cultivated in arid and semi-arid regions [4], where wheat yield improvement is constrained by salt stress. The low germination rate and frailty of seedlings under salt stress can result in plant death during winter and limited growth in the subsequent spring, increasing costs and decreasing yield [1,5,6]. Therefore, how salt stress causes a low germination rate and restricts seedling growth should be investigated, and approaches to alleviate salt stress should be identified to ensure the yield. Seed nano-treatment (treating seeds with nanomaterials) has recently emerged as a novel technique for enhancing the salt stress tolerance of crops [1,5,6]. For example, rapeseed treated with nanoceria (cerium oxide nanoparticles) have shown higher levels of α-amylase activity, reactive oxygen species (ROS) scavenging ability, and salicylic acid content under salt stress [1]. Nano-silicon increases the antioxidant enzyme system activity of barley seeds, enhancing their salt tolerance [6]. Moreover, seed nano-treatment is an economic approach [1]. The quantity of nanoceria applied to soil or leaves is three times less than that applied during seed nano-treatment [1]. Thus, treating seeds with nanomaterials could be a safe and cost-controllable choice for improving the salt tolerance of wheat.

Excessive ROS accumulation is a key factor causing plant death under salt stress [7]. Maintaining ROS homeostats enhances the salt tolerance of crops [8]. Nanozymes, which have enzyme-like activities [9], have been reported to effectively improve the salt tolerance of cotton [8], rapeseed [5], and rice [10] due to their antioxidant enzyme-like activity. Polyacrylic acid-coated manganese trioxide nanoparticles (PMOs) have been extensively studied as nanozymes for enhancing the tolerance of crops to abiotic stressors [11]. PMOs are safe nanozymes that can repair liver damage in animals [12]. PMOs reduce excessive ROS accumulation, enhancing the salt stress tolerance of cotton [11] and rape seedlings [13]. To date, no experiments have determined the impact of PMOs on the salt tolerance of monocotyledonous crops, especially using seed treatment methods. Monocotyledonous crops, such as wheat, serve as a major carbohydrate food source; therefore, the mechanism by which PMO treatment of monocotyledonous crop seeds enhances salt tolerance should be determined.

In terms of salt stress, the mechanisms by which nanotechnology enhances the salt tolerance of crops, in addition to maintaining ROS homeostasis, also include: (1) maintaining the K^+^/Na^+^ ratio balance in plant cells [14]; (2) regulating the amylase activity of seeds to enhance the germination rate under salt stress [1]; (3) regulating the hormonal balance in plants [15]; and (4) subjecting plants to stress acclimation by stimulating an early ROS burst [16]. However, to the best of our knowledge, it remains unknown whether these mechanisms increase crop yield under salt stress. Therefore, under the premise that nanozymes increase crop yield under salt stress, to address this knowledge gap, this study aims to investigate the mechanism of action of PMO, which will contribute to a deeper understanding of its impact on crop yield under salt stress.

Crop growth and development under salt stress have “trade-offs” [17]. Under salt stress, energy consumption is increased and energy generation is suppressed. When the energy level is very low, wheat seeds cease their activity [18]. Starch is the principal energy storage substance in wheat seeds, and the glucose generated from starch decomposition enters glycolysis and the tricarboxylic acid cycle, producing adenosine triphosphate [19]. Our previous study demonstrated that seeds treated with nanozymes have higher α-amylase and β-amylase contents under salt stress [1]. During seed germination, α-amylase converts starch into maltose and soluble sugars [20]. The increase in the soluble sugar content enhances the tolerance of crops to abiotic stressors, such as low temperature [21] and drought [22], and soluble sugar is a major energy source in seeds [23]. Alterations in metabolic processes (including the glycolysis pathway, tricarboxylic acid cycle, and pentose phosphate pathway) following enhanced starch decomposition by nanomaterials remain unclear. These processes are associated with the energy distribution in wheat seeds under salt stress. While our observations are consistent with the growth-defense tradeoff hypothesis, direct experimental validation—such as carbon/nitrogen partitioning analysis, stable isotope tracing of metabolic fluxes, or transcriptomic profiling—is required to conclusively demonstrate how this tradeoff is regulated under salt stress.

This study aimed to elucidate the physiological mechanism by which PMO-treated wheat seeds tolerate salt stress and to determine whether this tolerance is transient. By evaluating salt tolerance in wheat seeds treated with PMOs, we explored the mechanisms by which PMO mediates the “trade-off” between growth and resistance in wheat seeds under salt stress. We hypothesized that: (1) PMOs enhance the salt tolerance of wheat by maintaining ROS homeostasis; (2) PMOs regulate the balance of energy metabolism in wheat seeds, promoting their growth under salt stress; and (3) plants grown from PMO-treated seeds have a higher salt tolerance and yield.

## 2. Results

### 2.1. PMO Characterization

**Figure 1:**
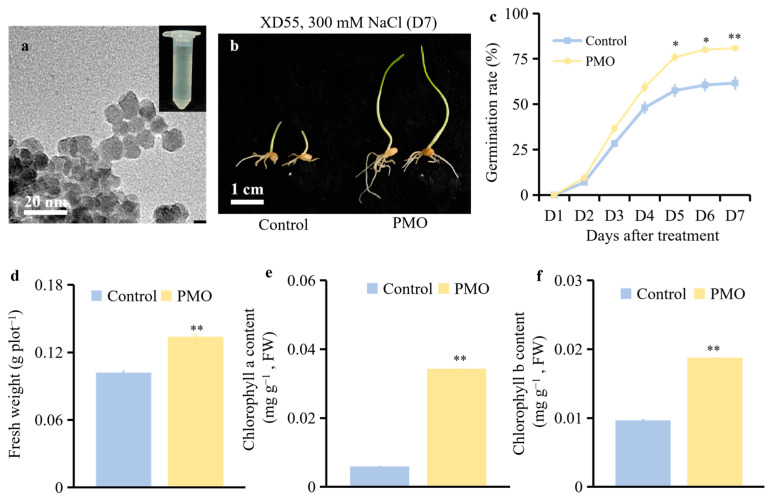
Transmission electron microscopy (TEM) image of polyacrylic acid-coated Mn_3_O_4_ nanoparticles (PMOs) and phenotypic performance of salt-stressed (300 mM NaCl, 7 days) wheat seeds with and without PMO treatment. (**a**) TEM image of PMOs. (**b**–**f**) Phenotypic performance (**b**), germination rate (**c**), fresh weight (**d**), and chlorophyll a (**e**) and chlorophyll b (**f**) contents of salt-stressed wheat seeds with and without PMO treatment. Mean ± SE (*n* = 6). * means *p* < 0.05, ** means *p* < 0.01.

The TEM images showed an average PMO core size of 9.2 ± 1.2 nm (Figure 1a), and HRTEM showed that the interplanar spacing was 0.488 nm, indicating (101) interplanar in Mn_3_O_4_ crystals (Figure S1a). Analysis using a dynamic light scattering instrument (Malvern Zetasizer Nano 90, Malvern, UK) showed that the average PMO size was 15.2 ± 1.2 nm based on the intensity (Figure S1b), and the zeta potential was −24.3 ± 2.1 mV (Figure S1c).

### 2.2. Soaking in PMO Improved Wheat Germination and Seedling Growth

The influence of soaking with PMO on the phenotype of wheat seeds grown under salt stress (300 mM NaCl) is shown in Figure 1b. Soaking seeds with PMO significantly increased wheat germination by 31.1% compared to the control group (61.7 ± 3.8 vs. 80.8 ± 1.7%, respectively) under salt stress (Figure 1c). Compared to the control group, soaking in PMO increased the fresh weight of wheat seeds by 31.4% (0.102 ± 0.002 vs. 0.134 ± 0.003 g box^−1^, respectively, under saline conditions; Figure 1d). However, compared to the non-salt stress control, seeds soaked or not soaked in PMO had limited germination and resulted in shorter seedlings under salt stress. Moreover, soaking in PMO significantly increased the chlorophyll a and chlorophyll b contents of wheat seeds by 400 and 100%, respectively, compared to the control group (0.006 ± 0.0 vs. 0.03 ± 0.0 mg g^−1^ for chlorophyll a and 0.01 ± 0.0 vs. 0.02 ± 0.0 mg g^−1^ for chlorophyll b; Figure 1e,f).

### 2.3. Soaking in PMO Reduced the Damaging Effects of ROS on Wheat Seeds and Increased the ROS Scavenging Ability

**Figure 2:**
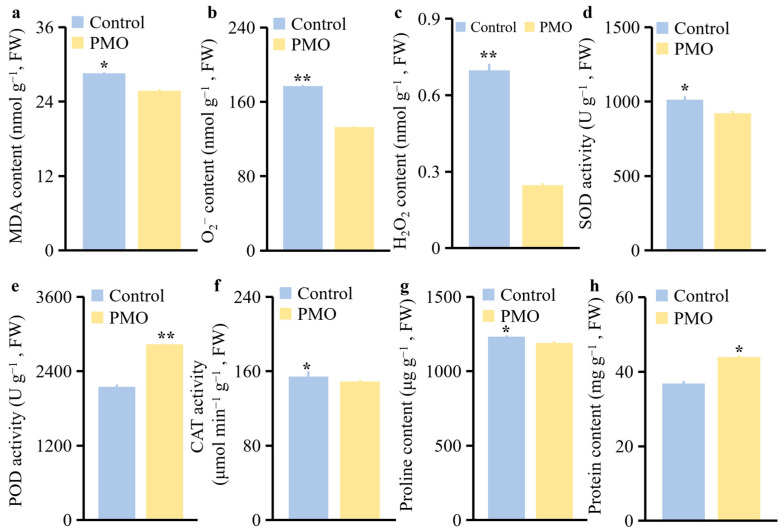
Reactive oxygen species (ROS), malondialdehyde (MDA), proline, protein content, and antioxidant enzyme activity of salt-stressed (300 mM NaCl, 7 days) wheat seeds with and without PMO treatment: MDA content (**a**), O_2_^−^ content (**b**), H_2_O_2_ content (**c**), superoxide dismutase (SOD, (**d**)), peroxidase (POD, (**e**)), and catalase activities (CAT, (**f**)), and proline (**g**) and protein contents (**h**). Mean ± SE (*n* = 3). * means *p* < 0.05, ** means *p* < 0.01.

Compared to the control group, soaking in PMO significantly decreased the contents of MDA (28.5 ± 0.2 vs. 25.8 ± 0.2 nmol g^−1^; Figure 2a), O_2_^−^ (176.9 ± 0.9 vs. 132.7 ± 0.7 nmol g^−1^; Figure 2b), and H_2_O_2_ (0.7 ± 0.0 vs. 0.2 ± 0.0 nmol g^−1^; Figure 2c) by 9.7, 25.0, and 64.7%, respectively, at 7 days after salt stress. A significant decrease in the SOD activity (1012.9 ± 24.3 vs. 922.3 ± 15.5 U g^−1^) and an increase in the POD activity (2148 ± 35.4 vs. 2833.6 ± 11.7 U g^−1^) were observed in seeds soaked in PMO compared to the control (Figure 2d,e), but no significant difference was observed in the CAT activity (154.1 ± 5.8 vs. 149.1 ± 1.6 μmol min^−1^ g^−1^; Figure 2f). Compared to the control group, the proline content significantly decreased (1232.3 ± 7.7 vs. 1190.8 ± 11.2 μg g^−1^) and the protein content significantly increased (36.8 ± 0.7 vs. 43.9 ± 0.5 mg g^−1^) in PMO-soaked seeds (Figure 2g,h).

**Figure 3:**
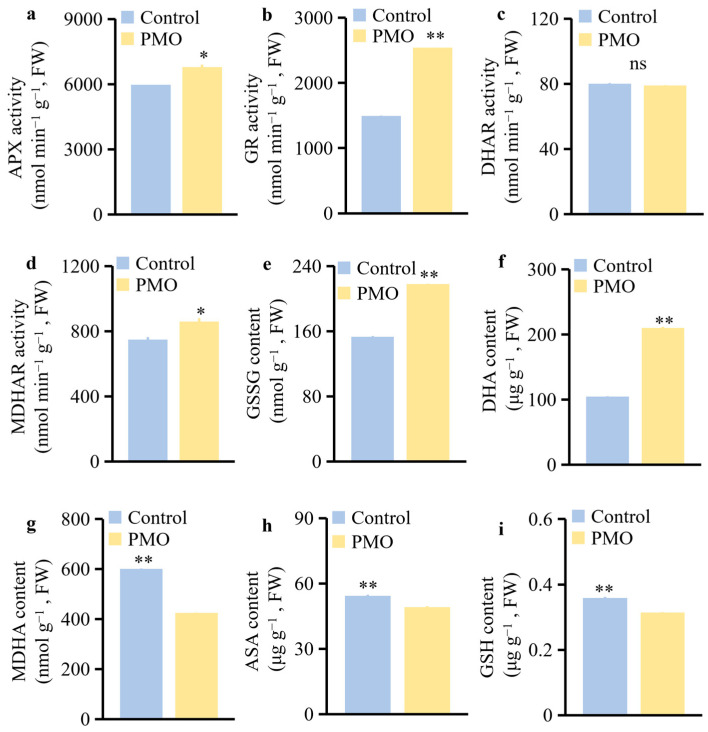
Key metabolite contents and enzyme activities in the ascorbic acid–glutathione (ASA-GSH) cycle in wheat seeds treated with and without PMOs under 300 mM NaCl (7 days): ascorbate peroxidase (APX, (**a**)), glutathione reductase (GR, (**b**)), dehydroascorbic acid reductase (DHAR, (**c**)), monodehydroascorbate reductase activities (MDHAR, (**d**)), and oxidized glutathione (GSSG, (**e**)), dehydroascorbate (DHA, (**f**)), monodehydroascorbate (MDHA, (**g**)), ascorbic acid (ASA, (**h**)), and glutathione contents (GSH, (**i**)). Mean ± SE (*n* = 3). * means *p* < 0.05, ** means *p* < 0.01. ns indicates no significant difference.

Compared to the control, 13.8, 70.2, and 14.6% increases in the APX (5970.0 ± 6.3 vs. 6793.0 ± 102.2 nmol min^−1^ g^−1^), GR (1493.6 ± 8.3 vs. 2541.4 ± 1.1 nmol min^−1^ g^−1^), and MDHAR activities (748.7 ± 14.2 vs. 858.1 ± 21.9 nmol min^−1^ g^−1^), respectively, were observed in PMO-soaked wheat seeds after 7 days under salt stress, but and no significant difference was observed in the DHAR activity (80.2 ± 0.3 vs. 79.1 ± 0.2 nmol min^−1^ g^−1^; Figure 3a–d). Soaking in PMO decreased the MDHA (600.8 ± 1.9 vs. 424.9 ± 1.0 nmol g^−1^), ASA (54.3 ± 0.5 vs. 49.1 ± 0.5 μg g^−1^), and GSH contents (0.4 ± 0.0 vs. 0.3 ± 0.0 μg g^−1^) in wheat seeds by 29.3, 9.6, and 12.7%, respectively, compared to the control group (Figure 3g–i).

### 2.4. PMO Modulates Glycolytic Pathways and the Tricarboxylic Acid Cycle in Wheat Seeds Under Salt Stress

**Figure 4:**
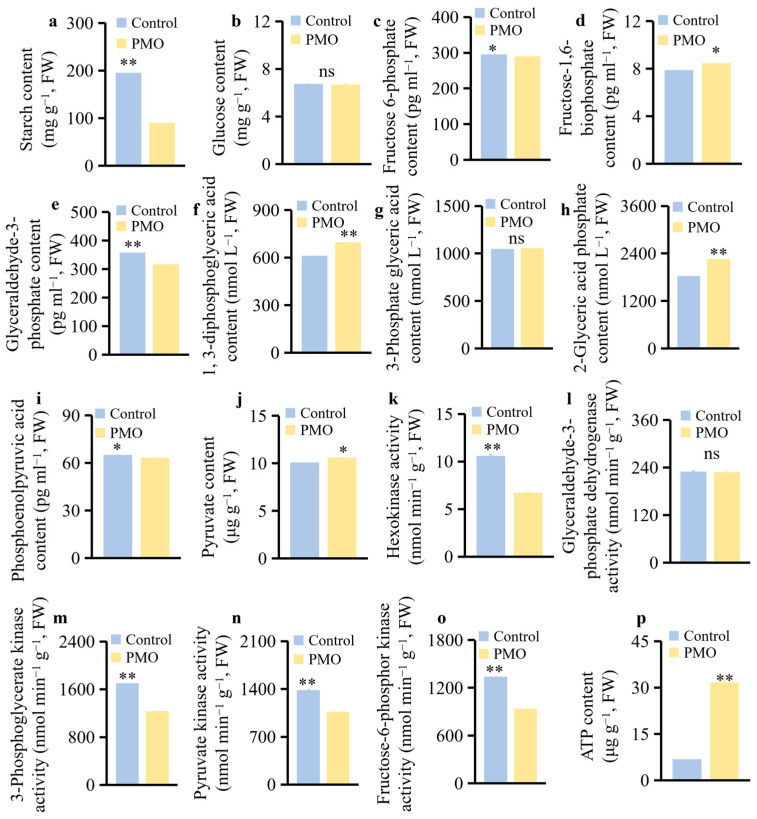
Key metabolite contents and enzyme activities in the glycolytic pathway in wheat seeds treated with and without PMOs under 300 mM NaCl (7 days): starch (**a**), glucose (**b**), fructose 6-phosphate (**c**), fructose-1,6-biophosphate (**d**), glyceraldehyde-3-phosphate (**e**), 1,3-diphosphoglyceric acid (**f**), 3-phosphate glyceric acid (**g**), 2-glyceric acid phosphate (**h**), phosphoenolpyruvic acid (**i**), pyruvate contents (**j**), hexokinase (**k**), glyceraldehyde-3-phosphate dehydrogenase (**l**), 3-phosphoglycerate kinase (**m**), pyruvate kinase (**n**), fructose-6-phosphor (**o**), and adenosine triphosphate (ATP, (**p**)). Mean ± SE (*n* = 3). * means *p* < 0.05, ** means *p* < 0.01. ns indicates no significant difference.

The effects of PMO on glycolysis and the tricarboxylic acid (TCA) cycle were further explored under salt condition. The contents of starch (195.3 ± 0.4 vs. 89.8 ± 0.8 mg g^−1^; Figure 4a), fructose 6-phosphate (295.6 ± 0.9 vs. 289.7 ± 1.1 pg mL^−1^; Figure 4c), glyceraldehyde-3-phosphate (357.7 ± 0.9 vs. 316.6 ± 0.8 pg mL^−1^; Figure 4e), and phosphoenolpyruvic acid (65.2 ± 0.2 vs. 63.3 ± 0.2 pg mL^−1^; Figure 4i) were significantly lower in PMO-treated wheat seeds when exposed to saline stress. However, the fructose-1,6-biophosphate (7.9 ± 0.0 vs. 8.5 ± 0.0 ng mL^−1^; Figure 4d), 1,3-diphosphoglyceric acid (611.6 ± 1.7 vs. 697.0 ± 1.6 nmol L^−1^; Figure 4f), 2-glyceric acid phosphate (1831.4 ± 6.7 vs. 2257.6 ± 6.4 nmol L^−1^; Figure 4h), and pyruvate (10.1 ± 0.0 vs. 10.6 ± 0.1 nmol min^−1^ g^−1^; Figure 4j) contents were more significantly increased in PMO-soaked seeds than in the control under salt stress. However, compared to the control group, soaking wheat seeds in PMO had no significant effect on the glucose (6.7 ± 0.0 vs. 6.7 ± 0.1 mg g^−1^; Figure 4b) or 3-phosphate glyceric acid content (1046.5 ± 2.7 vs. 1053.9 ± 2.5 nmol L^−1^; Figure 4g). Moreover, PMO treatment decreased the hexokinase (10.6 ± 0.2 vs. 6.7 ± 0.1 nmol min^−1^ g^−1^; Figure 4k), 3-phosphoglycerate kinase (1700.4 ± 2.5 vs. 1237.6 ± 11.9 nmol min^−1^ g^−1^; Figure 4m), pyruvate kinase (1379.7 ± 20.3 vs. 1069.9 ± 2.3 nmol min^−1^ g^−1^; Figure 4n), fructose-6-phosphokinase activities (1340.1 ± 1.8 vs. 939.1 ± 4.5 nmol min^−1^ g^−1^; Figure 4o) but had no significant impact on the glyceraldehyde-3-phosphate dehydrogenase activity (229.9 ± 3.6 vs. 227.7 ± 2.7 nmol min^−1^ g^−1^; Figure 4l). Soaking seeds in PMO also significantly increased the ATP (6.8 ± 0.1 vs. 31.8 ± 1.1 μg g^−1^; Figure 4p), NADH (11.3 ± 0.2 vs. 43.3 ± 0.7 nmol g^−1^; Figure S2a), NADPH (2.5 ± 0.4 vs. 7.8 ± 1.2 nmol g^−1^; Figure S2c), and NADP+ contents (5.3 ± 1.1 vs. 10.2 ± 2.7 nmol g^−1^; Figure S2d), but decreased the NAD+ content (1.9 ± 0.0 vs. 1.3 ± 0.0 nmol g^−1^; Figure S2b).

In the pentose phosphate pathway (PPP), soaking seeds in PMO under salt stress decreased the 6-gluconolactone phosphate (47.6 ± 0.1 vs. 42.1 ± 0.1 ng mL^−1^; Figure S2e) and 6-glucose phosphate dehydrogenase contents (30.0 ± 0.24 vs. 24.1 ± 0.1 nmol min^−1^ g^−1^; Figure S2h) but decreased the 6-phosphogluconate dehydrogenase content (179.1 ± 0.6 vs. 207.7 ± 1.2 U L^−1^; Figure S2f) compared to the control group. No significant changes were observed in the 6-gluconic phosphate content (282.4 ± 0.9 vs. 280.7 ± 0.6 pg mL^−1^; Figure S2g).

**Figure 5:**
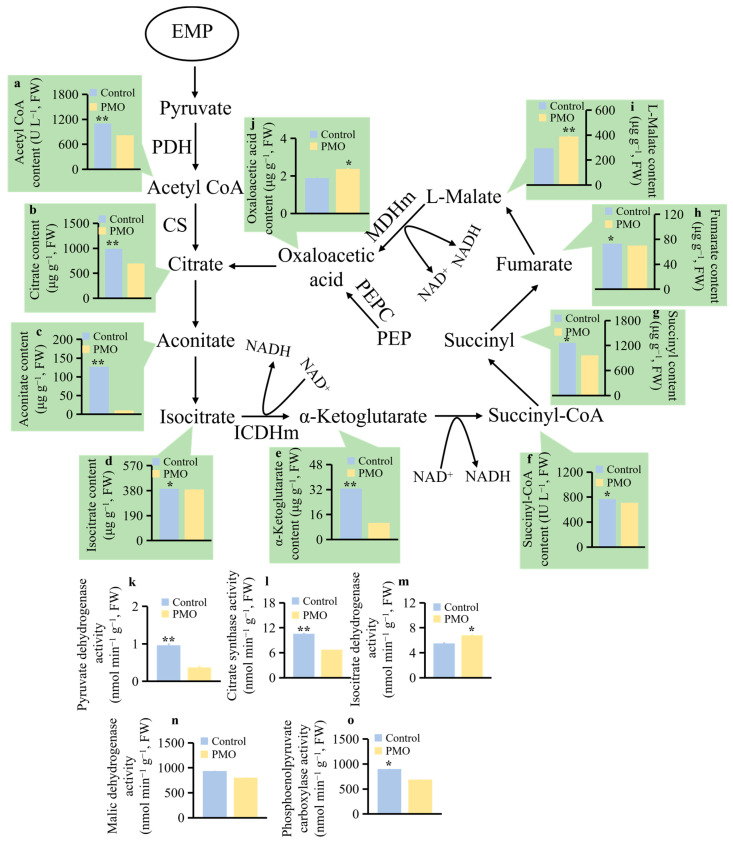
Key metabolite contents and enzyme activities involved in the tricarboxylic acid (TCA) cycle in wheat seeds treated with and without PMOs under 300 mM NaCl (7 days): acetyl CoA (**a**), citrate (**b**), aconitate (**c**), isocitrate (**d**), α-ketoglutarate (**e**), succinyl-CoA (**f**), succinyl (**g**), fumarate (**h**), L-malate (**i**), and oxaloacetic acid contents (**j**) and pyruvate dehydrogenase (**k**), citrate synthase (**l**), isocitrate dehydrogenase (**m**), malic dehydrogenase (**n**), and phosphoenolpyruvate carboxylase activities (**o**). Mean ± SE (*n* = 3). * means *p* < 0.05, ** means *p* < 0.01.

Compared to the control group, the acetyl CoA (1100.86 ± 5.48 vs. 818.44 ± 5.40 U L^−1^; Figure 5a), citrate (991.5 ± 4.3 vs. 693.24 ± 4.44 μg g^−1^; Figure 5b), aconitate (127.3 ± 0.0 vs. 10.6 ± 0.0 μg g^−1^; Figure 5c), isocitrate (393.8 ± 0.3 vs. 389.1 ± 1.0 μg g^−1^; Figure 5d), α-ketoglutarate (32.7 ± 0.1 vs. 10.6 ± 0.0 μg g^−1^; Figure 5e), succinyl CoA (768.7 ± 4.2 vs. 709.6 ± 4.1 IU L^−1^; Figure 5f), succinyl (1261.0 ± 6.1 vs. 965.2 ± 2.5 μg g^−1^; Figure 5g), and fumarate contents (73.2 ± 0.0 vs. 69.8 ± 0.1 μg g^−1^; Figure 5h) were significantly decreased and the L-malate (293.7 ± 3.2 vs. 388.0 ± 8.3 μg g^−1^; Figure 5i) and oxaloacetic acid contents (1.9 ± 0.0 vs. 2.4 ± 0.1 μg g^−1^; Figure 5j) were significantly increased in PMO-treated wheat seeds under salt stress. The pyruvate dehydrogenase (1.0 ± 0.1 vs. 0.4 ± 0.1 nmol min^−1^ g^−1^; Figure 5k), citrate synthase (10.6 ± 0.2 vs. 6.7 ± 0.1 nmol min^−1^ g^−1^; Figure 5l), malic dehydrogenase (933. 8 ± 8.2 vs. 800.4 ± 9.2 nmol min^−1^ g^−1^; Figure 5n), and phosphoenolpyruvate carboxylase activities (896.3 ± 6.3 vs. 687.2 ± 3.6 nmol min^−1^ g^−1^; Figure 5o) were significantly decreased and the isocitrate dehydrogenase activity (5.5 ± 0.2 vs. 6.8 ± 0.2 nmol min^−1^ g^−1^; Figure 5m) was increased in PMO-soaked seeds compared to the control group.

### 2.5. Soaking in PMO Enhanced the Salt Tolerance and Yield of Wheat Seedlings

**Figure 6:**
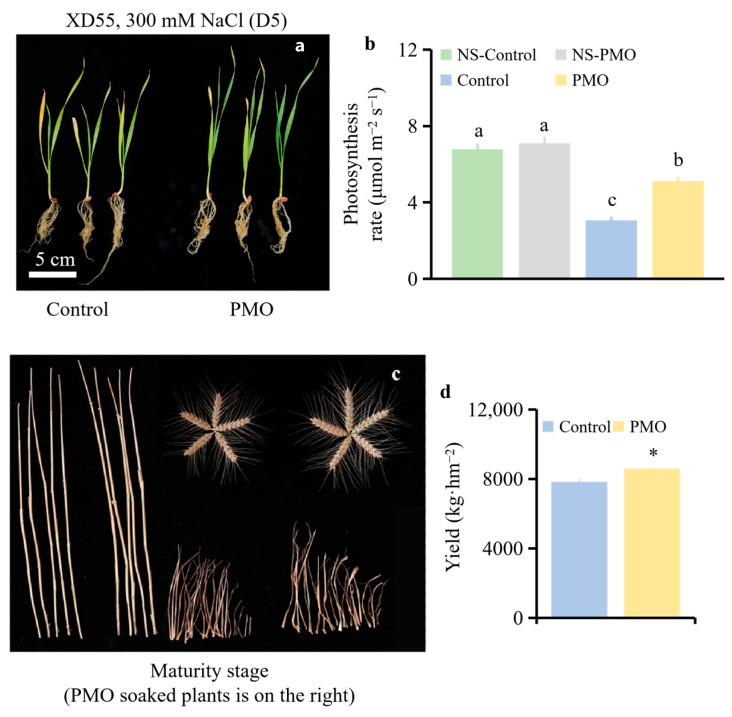
Phenotypic performance and yield of salt-stressed wheat with and without PMO soaking. (**a**) Phenotypic performance of wheat seedlings grown from seeds with and without PMO soaking. (**b**) Photosynthesis rate of wheat seedlings grown from seeds with and without PMO soaking under salt or normal condition (*n* = 6). (**c**) Phenotypic performance of mature stage wheat grown from seeds with and without PMO soaking. (**d**) Yield of wheat grown from seeds with and without PMO soaking (*n* = 5). Mean ± SE. * means *p* < 0.05. Different lowercase letters indicate *p* < 0.05.

Compared to wheat seedlings grown without PMO treatment, seedlings treated with PMO showed better phenotypic performance (Figure 6a) and a significantly higher fresh weight (Figure S3b) under 300 mM NaCl. The control and PMO-treated seedlings in the no-salt stress control exhibited a significantly higher fresh weight (0.56 ± 0.02 and 0.55 ± 0.02 g; Figure S3b) and SPAD value (40.40 ± 0.45 and 41.41 ± 0.49; Figure S3d) compared to those under salt stress. There were no visible differences in the canopy, but the roots of the seedlings without salt stress were more developed (Figure S3a,c). Under 300 mM NaCl, the SPAD value in PMO-treated seedlings was increased (22.75 ± 0.46) compared to the control group (20.73 ± 0.52; Figure S3d). Additionally, under 300 mM NaCl, PMO-treated wheat seedlings had a significantly higher plant height than the control group (Figure S3c).

After 5 days of growth, wheat seedlings without salt stress had a significantly higher carbon assimilation rate (6.8 ± 0.3 vs. 7.1 ± 0.4 µmol m^−2^ s^−1^; Figure 6b), transpiration rate (0.003 ± 0.000 vs. 0.003 ± 0.000 mol m^−2^ s^−1^; Figure S3e), and stomatal conductance (0.2 ± 0.0 vs. 0.2 ± 0.0 mol m^−2^ s^−1^; Figure S3f) but showed no difference in the intercellular CO_2_ concentration (310.1 ± 9.4 vs. 322.1 ± 2.8 µmol mol^−1^; Figure S3g) compared to seedlings grown under salt stress. Under salt stress, PMO-treated seedlings presented a higher carbon assimilation rate (3.1 ± 0.2 vs. 5.1 ± 0.3 µmol m^−2^ s^−1^; Figure 6b), transpiration rate (0.002 ± 0.000 vs. 0.002 ± 0.000 mol m^−2^ s^−1^; Figure S3e), and stomatal conductance (0.1 ± 0.0 vs. 0.1 ± 0.0 mol m^−2^ s^−1^; Figure S3f) but showed no significant difference in the intercellular CO_2_ concentration (327.7 ± 3.1 vs. 310.3 ± 5.0 µmol mol^−1^ Figure S3g) compared to the control group.

To evaluate the effect of soaking wheat seeds with PMO on yield in saline land, we carried out a field experiment from 2024 to 2025. Wheat soaked in PMO had larger ears and leaves (Figure 6c) and thus achieved a higher yield (7834.9 ± 188.2 vs. 8601.7 ± 132.8 kg ha^−1^, Figure 6d). Although PMO-treated wheat showed no significant increase in density (443.2 ± 12.4 × 104 vs. 448.4 ± 13.2 × 104 plants ha^−1^, Figure S3h) and a lower 1000-grain weight (51.2 ± 0.3 vs. 49.1 ± 0.2 g, Figure S3i), it had more grains (16.2 ± 0.3 vs. 17.6 ± 0.5, Figure S3j) and spikes (485.4 ± 11.7 vs. 519.6 ± 8.0 plant ha^−1^, Figure S3k) compared to the control group.

## 3. Discussion

### 3.1. PMO Mitigates the Negative Effects of Salt Stress on Wheat Seeds and Seedlings

The Mn_3_O_4_ nanozyme was initially reported to possess antioxidant efficacy and to eliminate inflammation in mice [24], demonstrating its intrinsic enzyme-mimicking activity in animal systems; it also alleviates drug-induced liver injury in animals by mitigating ferroptosis and is non-toxic to healthy humans within a reasonable concentration range [12]. Subsequently, the application of Mn_3_O_4_ nanozyme was extended to plants, where it was employed to enhance the salt tolerance of cucumbers [25], and Liu et al. [11] demonstrated that PMO improved the salt tolerance of cotton. In both of these plant studies, PMO was applied via foliar spray, and the results showed that foliar application effectively reduced ROS levels and the Na^+^/K^+^ ratio in leaves, thereby enhancing crop salt tolerance [11,13]. Analogous to our findings, PMO was characterized as spherical particles with a size of 9.2 ± 1.2 nm, a zeta potential of −24.3 ± 2.1 mV, and a crystal plane spacing of 0.488 nm, and exhibited excellent antioxidant capacity (Figure S1a–c). Notably, while foliar application of PMO has been relatively well studied in plants, research on seed-based applications remains limited; to date, only one study has shown that PMO-treated germinating corn seeds have enhanced drought tolerance [26]. Thus, the role of PMO on crop seeds under salt stress remains undefined, and in addition to the antioxidant enzyme system, whether there are other mechanisms by which PMO regulates ROS homeostasis in crops and the mechanism by which it regulates seed development should be investigated.

Salt stress has a negative impact on wheat seed germination [27], particularly in Xinjiang, which is a major wheat-growing region. The osmotic stress induced by salt stress can impede water absorption in seeds, hindering seed germination or reducing the germination rate [28]. Studies showing a reduced germination capacity, biomass accumulation, and chlorophyll content and inhibited growth due to salt stress have been reported in rapeseed [1], sorghum [29], sunflower [30], and soybean [31]. Similar results were obtained in our study. Compared to the no salt control, the low daily growth resulting from 300 mM NaCl stress reduced the fresh seed weight and chlorophyll content (Figure 1d–f). This indicates that, like other species, wheat seeds also demonstrate salt sensitivity, as evidenced by the decreased germination rate and seed growth, even for varieties widely promoted in southern Xinjiang. In our study, seeds soaked in PMO performed better under salt stress compared to the control, with increases in the germination rate, fresh weight, and chlorophyll content by 31.4, 31.1, and 240.4%, respectively (Figure 1c–f), but only reaching 80% of the values obtained in the no salt control (Figure S1e). These results are analogous to those of previous studies using nanozymes for seed treatment. For example, Se-doped carbon dots and nanoceria nanozymes enhance the growth and photosynthetic pigment synthesis of rape seeds under salt stress [1,5]. Nanoceria initiation improves the salt tolerance of rape seeds during the germination period. Zinc oxide nanoparticles increase the germination rate of rapeseed and wheat [32] under salt stress. Silver nanoparticles [32,33] and zero-valent iron nanoparticles [34] enhance the germination rate of rice under salt stress. PMO has also been reported to enhance the salt tolerance of soybean seeds [35]. Previous research as well as our results suggest that treating crop seeds with nanomaterials is an effective approach for improving their salt tolerance, but it cannot restore them to the level obtained without salt stress. Therefore, analysis of the mechanism by which nanomaterials enhance seed germination will assist us in continuously enhancing the effects of nanomaterials to improve crop salt tolerance.

Ensuring wheat growth during the seedling stage prior to overwintering constitutes an essential measure for guaranteeing yield [36]. Concurrently, the seedling stage is a significant phase that is prone to the hazards of salt stress. Mitigating the inhibition of wheat seedling growth caused by salt stress is a crucial measure for ensuring yield. We found that wheat seeds at the two-leaf stage with and without soaking in PMO under no salt stress showed no significant differences in phenotype or photosynthesis (Figure S3a,e). After imposing salt stress on wheat at the seedling stage, seedlings pre-treated with PMO exhibited better salt tolerance and photosynthetic rates than those in the control group (Figure S3e). Another study in which seeds were treated with PAA@Se-CDs found similar results [5]. A previous study has suggested that the increase in ROS triggered by the nanozyme enhances the salt tolerance of crops [37], which could explain our results. Salt stress in our experiment was imposed on wheat at the two-leaf stage, whereas continuous salt stress was applied starting from the seed stage in the PAA@Se-CD study. Our field experiments confirmed that soaking seeds in PMO had a promoting effect on wheat yield under saline–alkali stress, which was mainly achieved by increasing photosynthesis and the number of grains and spikes (Figure 6b and Figure S3j,k). However, the wheat planting density did not increase, which was inconsistent with our expectations, which could be because we adopted the strip sowing method during planting, ensuring a certain number of seedlings. This shows that soaking seeds in nanozymes can endow crops with salt tolerance for a relatively long time.

### 3.2. Stimulation of Antioxidant Enzyme Systems After Soaking in PMO Is a Key Factor for Improving Wheat Germination Under Salt Stress

Salt stress causes a metabolic imbalance in plants and induces ROS accumulation [8]. ROS have a dual function in plants, and excessive ROS accumulation under salt stress is regarded as a crucial factor contributing to crop growth inhibition [7]. Under salt stress, excessive ROS can damage proteins, DNA, membranes, and other biomolecules in plant cells [38]. The content ROS and the extent of oxidative damage are considered significant indicators for assessing the salt tolerance capacity of crops [39]. In this study, the O_2_^−^, H_2_O_2_, and MDA contents in wheat seeds treated with PMO decreased significantly compared to the control group (Figure 2a–c). This suggests that PMO mitigated the oxidative damage endured by wheat seeds under salt stress. The SOD and CAT activities also decreased, possibly because SOD primarily converts O_2_^−^ to H_2_O_2_, while CAT scavenges H_2_O_2_ (Figure 2d–f).

After 7 days of salt stress, the proline content in PMO-treated wheat seeds was lower than that in the control (Figure 2g). Proline contributes to salt tolerance through three main mechanisms [40]: (1) it acts as an osmotic regulator, enhancing cellular water uptake; (2) it functions as a compatible solute and reactive oxygen species (ROS) scavenger, effectively neutralizing superoxide anions and hydroxyl radicals; and (3) it provides energy via its catabolism to support crop growth and development. In our study, the decreased proline content after PMO treatment indicated antioxidant and energy metabolism, while the maintenance of the osmotic potential was regulated by the increase in the soluble protein content (Figure 2g). These results are in line with previous studies, suggesting that PMO assists plants in resisting stress, such as salinity, by scavenging ROS [11]. Our findings further support the utilization of nanozymes for maintaining ROS homeostasis and enhancing the salt tolerance capacity of crops in nano-agriculture and validate the potential of nanozymes for improving the salt tolerance of wheat seeds using seed treatment applications. This nano-biotechnological approach offers a technical reserve for wheat growers, particularly in regions constrained by soil salinization, to lower costs and boost yields. Mn-based nanomaterials, including Mn_3_O_4_, have demonstrated favorable biocompatibility and good biosafety profiles in various biological systems [41]. For instance, Mn_3_O_4_ nanoparticles at appropriate concentrations exhibit no cytotoxicity and effectively scavenge ROS [24]. However, Considering safety concerns, bioaccumulation and the effects on non-target organisms should be evaluated when applying nano-biotechnology in food crops.

The main mechanisms by which nanozymes maintain ROS homeostasis in crops include the following: (1) enhancing the antioxidant enzyme systems of crops [25]; (2) directly removing excessive ROS [14]; and (3) enhancing the antioxidant capacity of crops through early stress training (such as inducing ROS production) [42]. Maintaining the ROS balance in crops through the first and third pathways requires energy consumption. A growing body of literature supports the concept that nanoparticle seed priming induces a mild and transient oxidative stress that serves as a signaling cue to activate downstream defense responses [43]. During imbibition, low concentrations of nanoparticles can induce transient ROS production through combined nanoparticle and ion effects, together with moderate thiol-based redox shifts acting as developmental signals. These signals activate antioxidant systems, mitogen-activated protein kinase cascades, and hormone-dependent pathways that coordinate metabolic activation and membrane repair. This controlled redox modulation may establish a primed physiological state that improves tolerance to abiotic stress during early seedling establishment. To the best of our knowledge, no studies have reported the regulation of the non-enzymatic antioxidant system in crops using nanozymes.

### 3.3. PMOs Regulate Sugar Decomposition to Provide Energy for the Non-Enzymatic Antioxidant System

In plant cells, in addition to H_2_O_2_ and O_2_^−^, which were determined in this study, the ROS induced by abiotic stress also encompass ·OH and ^1^O_2_, which cannot be scavenged by any known antioxidant enzyme system [44]. Among the 4 ROS, ·OH is regarded as the most destructive and is generated through the Fenton reaction between H_2_O_2_ and transition metals, such as Fe^2+^ [45]. To mitigate ·OH toxicity, plants have evolved non-enzymatic antioxidant systems, such as the ASA-GSH cycle [45]. To the best of our knowledge, no studies have reported the regulation of the ASA-GSH cycle by treating seeds with nanozymes.

The ASA-GSH cycle is a non-enzymatic antioxidant system present in the chloroplasts and cytoplasm of plant cells that consists of ASA, MDHA, GSH, DHA, GSSG, MDHAR, GR, DHAR, and APX. With NADH and NADPH serving as energy sources, it effectively scavenges ROS, including H_2_O_2_ and ·OH [46,47]. In our study, the DHA and GSSG contents and GR, MDHAR and APX activities increased in wheat seeds soaked in PMO, whereas the GSH and ASA contents decreased (Figure 3). ASA is a vitamin that directly neutralizes hydroxyl radicals and generates MDHA [38]. Under the catalysis of MDHAR, MDHA is reverted to ASA through the energy provided by NADH, generating NAD+. Additionally, ASA also participates in the process through which APX decomposes H_2_O_2_ and generates MDHA. The intensification of MDHAR and APX activities fortifies the process by which ASA eliminates ROS. The energy to reduce MDHA and generate ASA (with NADH as the donor) is provided by isocitrate dehydrogenase in the TCA cycle and glyceraldehyde-3-phosphate dehydrogenase in the Embden Meyerhof pathway. Concurrently, ASA can also directly react with superoxide anions to yield DHA, but the DHAR activity does not increase, which might explain the increase in the DHA content in PMO-treated wheat seeds (Figure 3f). The decline in the ASA content might be the result of the lack of an increase in the DHAR activity because ASA chiefly participates in the direct elimination of O_2_^−^ (Figure 2b and Figure 3h). These results account for the decrease in SOD activity and the superoxide anion content.

In animal cells, cell death resulting from excessive hydroxyl radical accumulation is designated as ferroptosis [48]. A significant characteristic of ferroptosis is the low content of GSH, which serves as a crucial hydroxyl radical scavenger [49]. The results of this study indicate that the GSH content decreased in PMO-treated wheat seeds, but the GSSG content and GR activity significantly increased (Figure 3b,e,i). This could be attributed to the involvement of GSH in the hydroxyl radical scavenging process in cells. The increase in GR activity may replenish GSH, and the energy used by GR to reduce GSSG to GSH originates from NADPH. The increase in 6-phosphogluconate dehydrogenase activity suggests that the impetus through which GR reduces GSSG stems from the PPP pathway (Figure S2).

### 3.4. Coordination of Energy Allocation Between Plant Growth and Defense Is the Key for Improving the Salt Tolerance of Crops

The life activities of crops are underpinned by energy, but energy is finite. In seeds, the energy for life activities primarily stems from internally stored organic substances. For example, the energy for life activities in wheat seeds originates from the decomposition of stored starch [50]. Consequently, the energy accessible to seeds is even more restricted. Plants under salt stress require more energy than usual to maintain their salt-tolerance mechanisms. To maintain normal growth, plants must balance energy supply and demand during salt stress to sustain growth and development [18]. The “growth–defense tradeoff” hypothesis states that the cost for plants to tolerate external stress is considerable [17]. According to this hypothesis, crops under normal circumstances diminish their own tolerance to stress because investing energy in defense significantly curtails the resources for growth and reproduction. Through the analysis of existing data for 200 plant species, Giolar et al. found that wild-type plants exhibited a “growth–defense tradeoff”, but this phenomenon was absent in domesticated crops [17]. This might be due to trait loss during the crop domestication process, as artificial cultivation provides a superior growth environment. Nevertheless, such loss may result in irreversible crop death under domestication conditions. Thus, maintaining the balance in energy allocation between defense and growth under salt stress can enhance the salt tolerance of crops.

Glycolysis involves oxidizing glucose derived from starch into pyruvate, and the TCA cycle subsequently decarboxylates pyruvate to generate reductants, such as NADH [49,51]. NADH drives the ASA-GSH cycle or enters mitochondria to produce ATP [52]. Under salt stress, the 6-gluconolactone phosphate content decreased significantly in salt-tolerant soybean varieties, which is in accordance with our results (Figure S2e) [53]. The content of pyruvate, the final product of the glycolysis pathway, in PMO-treated wheat seeds increased (Figure 4j), which is consistent with observations in wild barley under salt stress [54]. This suggests that PMO treatment results in a “growth–defense tradeoff” in cultivated wheat and that the glycolysis pathway plays a crucial role in this process. Furthermore, a study in sugar beets has shown that the contents of glycolysis metabolites increase in response to the augmented energy demand under salt stress [55]. In our study, the abundance of glycolysis metabolites in PMO-treated wheat seeds decreased compared to the control group (Figure 4), suggesting that the energy demand for salt stress tolerance declined. The PPP pathway connects mitochondrial respiration and energy metabolism and generates NADPH. In the present study, the activity of 6-phosphogluconate dehydrogenase, a key enzyme in the PPP pathway that generates NADPH, increased in the PMO group, providing the impetus by which GR reduces GSSG to GSH (Figure 3 and Figure S2f). Thus far, the mechanism through which salt stress influences the PPP pathway has not been explicitly reported, and its role in the “growth–defense tradeoff” in crops remains to be addressed.

Pyruvate decomposition in the TCA cycle generates energy, which is the main source for plant life activities. The TCA cycle interconverts malate and homogentisate through a series of reactions and generates NADH, providing energy for plant growth [56]. Research has shown that the abundance of intermediate products in the TCA cycle of salt-tolerant rice varieties is decreased. The decreased organic acids are utilized for amino acid synthesis, which might explain the faster development of PMO-treated wheat seeds [57]. The contents of TCA cycle intermediates under salt stress vary significantly among crop species. In our study, soaking in PMO promoted crop growth by decreasing the TCA cycle activity (Figure 5), which might be attributed to the tradeoff between wheat growth and stress tolerance mediated by PMO. In our findings, the contents of key enzymes involved in the TCA cycle, except for isocitrate dehydrogenase, decreased in PMO-treated wheat seeds (Figure 5m). This suggests that the wheat seeds in the PMO group do not require excessive energy to maintain salt tolerance mechanism, such as antioxidant function. The cause of this change might be related to the ROS scavenging ability of PMO. The increase in isocitrate dehydrogenase activity supplies energy for the ASA-GSH cycle.

## 4. Materials and Methods

### 4.1. PMO Synthesis and Characterization

Briefly, 0.425 g MnSO_4_·H_2_O (M7634, Sigma Aldrich, St. Louis, MO, USA) and 4.5 g poly(acrylic) acid (323667, Sigma Aldrich, St. Louis, MO, USA) were dissolved in 2.5 and 5 mL ddH_2_O, respectively. The solutions were then vortexed at 2000× *g* for 15 min. The mixture was subsequently added dropwise to 15 mL of 30% ammonium hydroxide solution (Sigma Aldrich) in a 50-mL glass beaker while stirring at 500× *g* for 24 h at 25 °C in a fume hood. After 24 h, the solution was transferred to a 50-mL reaction kettle and placed in a hydrothermal oven at 120 °C for 24 h. After hydrothermal treatment, the solution was centrifuged at 6000× *g* for 1 h. The obtained supernatant was dialyzed using a 10-kDa dialysis bag for 24 h. The synthesized PMO concentration was calculated using the heat-weight method. The PMOs were stored at 4 °C for further use.

Transmission electron microscopy (TEM) and high-resolution TEM (HRTEM) images were captured on a microscope (FEI Talos F200C, Hillsboro, OR, USA) with an accelerating voltage of 200 kV. The TEM samples were prepared by adding them dropwise to a copper mesh. The size and zeta potential of the nanoparticles were determined using a Malvern Zetasizer Nano 90 (Malvern, UK).

### 4.2. Seed Materials, Seed Soaking, Stress Treatments, Growth Conditions, and Field Experiment

Wheat variety ‘Xindong 55’ (XD 55) was used in this experiment. Moreover, through preliminary concentration screening experiments, 200 mg L^−1^ was identified as the optimal concentration for subsequent experiments, while ddH_2_O alone served as the control group. Seeds were immersed in either PMO or ddH_2_O solution. Conical flasks containing the seeds and soaking solution were placed on a shaker (100× *g*) with constant agitation for 3 h, with a seed to solution ratio of 1:5 (*w*:*v*). After soaking for 3 h, the seeds were rinsed with ddH_2_O. The soaked seeds were sown in polyethylene boxes (10 cm × 10 cm × 3 cm length × width × height), with 30 seeds per box. The boxes contained uniform SiO_2_ particles (0.5 cm in height) moistened with 15 mL of 300 mM NaCl solution or ddH_2_O. Every third day, 5 mL of salt solution or ddH_2_O was added to the boxes. The boxes were exposed to a photoperiod of 14 h light (300 μmol m^−2^ s^−1^) and 10 h dark at 28 ± 1 and 20 ± 1 °C, respectively. The germination rate was recorded daily, and the germination experiment was terminated 7 days after sowing. The fresh weight was recorded immediately.

To ensure rigorous biological replication, the experiment was conducted using multiple independent seed batches. For biochemical measurements, pooled samples were prepared by combining equal amounts of tissue from multiple independent batches into one composite sample. Each composite sample was treated as one biological replicate, and a total of three such composite samples were obtained for each treatment (*n* = 3). All samples were immediately frozen in liquid nitrogen for subsequent analyses.

Wheat seeds soaked for 3 h in ddH_2_O or PMO were sown in pots (10 cm × 10 cm) filled with a standard soil mix. Plants were grown in a growth room under the following settings: 200 μmol m^−2^ s^−1^ photosynthetic active radiation (PAR), 28 ± 1 °C (day) and 25 ± 1 °C (night), 70% relative humidity, and 14/10 h photoperiod. After wheat plants reached the two-leaf stage, 15 mL of ddH_2_O or 300 mM NaCl solution was applied and left to soak for 7 days. The fresh weight, SPAD value, and plant height were recorded at 7 days after treatment.

A field experiment was conducted in 2024–2025. Wheat seeds soaked for 3 h in ddH_2_O or PMO were sown in a field with an 8.3‰ salt content in Alar, China. The soil at the test site was loamy. Analyses of the 0–20 cm soil layer revealed a soil organic matter content of 7.18 g·kg^−1^, alkaline hydrolyzable nitrogen content of 25.6 mg·kg^−1^, available phosphorus content of 20.1 mg·kg^−1^, and available potassium content of 140.7 mg·kg^−1^. The preceding crop was cotton. Drip irrigation was applied throughout the growing season with integrated water-fertilizer management, and the total precipitation during the experimental period in 2024 was 56.8 mm. Pest and disease control, as well as weed removal, were performed according to standard local agricultural practices. There were 3 replications for each treatment, and each replication had an area of 30 m^2^ (5 m × 6 m). The final yield was determined from 3 replications for each of the two treatments.

### 4.3. Measurement of the Carbon Assimilation Rate and Chlorophyll Content

Photosynthetic performance was measured using a photosynthesis meter Li-6800 (LI-COR Inc., Lincoln, NE, USA). In the leaf chamber, the CO_2_ concentration was adjusted to 400 μmol m^−2^ s^−1^ using a CO_2_ mixer, and the leaf temperature and humidity were maintained at 25 °C and 70%, respectively. Other parameters were uncontrolled to be consistent with a stable growth environment. The gas exchange parameters were recorded after equilibration to a steady state.

Samples were mixed with a solution containing acetone and ethanol (1:1) in the dark on a shaker (50× *g*) for 24 h. After centrifugation at 4000× *g* for 10 min, the supernatant was collected. The absorbance of the supernatant was measured at 644 nm for Chl a and 662 and 440 nm for Chl b using a spectrophotometer.

### 4.4. Oxidative Damage, Soluble Protein Content, and Enzymatic and Non-Enzymatic Antioxidant Activity Assays

At 7 days after sowing, the seedlings in the box were washed with ddH_2_O, surface dried with blotting paper, and dissected into roots and shoots. After weighing 0.1 g of root and shoot tissues, the samples were flash frozen in liquid nitrogen and transferred to a −80 °C freezer until further use.

The protein, malondialdehyde (MDA), O_2_^−^, H_2_O_2_, proline, oxidized glutathione (GSSG), dehydroascorbic acid (DHA), monodehydroascorbic acid (MDHA), ascorbic acid (ASA), and glutathione (GSH) contents and peroxidase (POD), catalase (CAT), superoxide dismutase (SOD), ascorbate peroxidase (APX), glutathione reductase (GR), dehydroascorbate reductase (DHAR), and monodehydroascorbate reductase (MDHAR) activities were determined using assay kits purchased from Suzhou Mengxi Bioengineering Institute Co., Ltd. (Suzhou, Jiangsu, China). Three biological replicates were used to quantify the ROS content and enzymatic and non-enzymatic antioxidant activities.

### 4.5. Sugar, Starch, and Key Metabolic Enzyme Contents

Starch, glucose, fructose 6-phosphate, fructose-1,6-biophosphate, glyceraldehyde-3-phosphate, 1, 3-diphosphoglyceric acid, 3-glyceric acid phosphate, 2-glyceric acid phosphate, phosphoenolpyruvic acid, pyruvate, nicotinamide adenine dinucleotide (NADH), oxidized NADH (NAD+), nicotinamide adenine dinucleotide phosphate (NADPH), oxidized NADPH (NADP+), adenosine triphosphate (ATP), 6-gluconolactone phosphate, 6-gluconic phosphate, acetyl CoA, citrate, aconitate, isocitrate, α-ketoglutarate, succinyl-CoA, succinyl, fumarate, L-malate, and oxaloacetic acid contents and hexokinase, glyceraldehyde-3-phosphate dehydrogenase, 3-phosphoglycerate kinase, pyruvate kinase, fructose-6-phosphor kinase, 6-phosphogluconate dehydrogenase, 6-glucose phosphate dehydrogenase, pyruvate dehydrogenase, citrate synthase, isocitrate dehydrogenase, malic dehydrogenase, and phosphoenolpyruvate carboxylase activities were determined using assay kits purchased from Suzhou Mengxi Bioengineering Institute Co., Ltd. (Suzhou, Jiangsu, China). Three biological replicates were used to quantify the contents of sugar, starch, and key metabolic enzymes.

### 4.6. Statistical Analysis

All data were expressed as mean ± standard deviation (SD) of three independent biological replicates (*n* = 3), and statistical analyses were performed using SPSS 12.0 software, with graphs generated using Microsoft Excel 2016. Prior to parametric testing, the normality of data distribution was assessed using the single-sample Kolmogorov–Smirnov test, and the homogeneity of variances among groups was evaluated using Levene’s test. For comparisons involving only two groups, the independent sample *t*-test (two-tailed) was employed, while for comparisons involving three or more independent groups, one-way ANOVA was performed; when a significant main effect was detected (*p* < 0.05), Duncan’s multiple range test was used for post hoc multiple comparisons to control the comparison-wise error rate. In cases where Levene’s test indicated significant heterogeneity of variances (*p* < 0.05), data were appropriately transformed prior to ANOVA, or the non-parametric Kruskal–Wallis test was applied as an alternative. A probability value of *p* < 0.05 was considered statistically significant in all tests.

## 5. Conclusions

Overall, salt stress induces ROS accumulation and energy consumption, hindering wheat growth. Soaking seeds in PMOs promotes wheat germination under salt stress by maintaining ROS stability and enhancing antioxidant system activity. Furthermore, soaking seeds in PMOs regulates sugar metabolism, promoting growth and maintaining yield. In summary, soaking seeds in PMOs can balance the energy consumed by wheat seeds for stress tolerance and growth, enhancing the germination rate, seedling growth, and yield formation under salt stress. Our research breaks through the traditional research approach on the salt tolerance of crop seedlings at an early stage. Starting from the formation of yield and energy consumption, the results offer a new perspective for the study of nano-biotechnology.

## Data Availability

Data will be made available upon request.

## Supplementary Materials

The following supporting information can be downloaded at https://www.mdpi.com/article/10.3390/plants15142124/s1, Figure S1: Characteristics of PMOs and phenotypic performance of wheat seeds with and without PMO soaking under no-salt conditions (7 days); Figure S2: Key metabolite contents and enzyme activities involved in the pentose phosphate pathway in wheat seeds treated with and without PMOS under 300 mM NaCl (7 days): NADH content; Figure S3: Phenotypic performance and photosynthesis parameters of wheat seedlings grown from seeds with and without PMO soaking; Figure S4: Representative photographs of wheat germination communities in the PMO-treated and control groups, taken on the 3rd and 5th days after soaking.

## Abbreviations

The following abbreviations are used in this manuscript:

ROS	reactive oxygen species
PMO	polyacrylic acid-coated Mn_3_O_4_ nanoparticles
TEM	transmission electron microscopy
HRTEM	high-resolution transmission electron microscopy
MDA	malondialdehyde
GSSG	oxidized glutathione
DHA	dehydroascorbic acid
MDHA	monodehydroascorbic acid
ASA	ascorbic acid
GSH	glutathione
POD	peroxidase
CAT	catalase
SOD	superoxide dismutase
APX	ascorbate peroxidase
GR	glutathione reductase
DHAR	dehydroascorbate reductase
MDHAR	monodehydroascorbate
NADH	nicotinamide adenine dinucleotide
NAD+	oxidized NADH
NADPH	nicotinamide adenine dinucleotide phosphate
NADP+	oxidized NADPH
ATP	adenosine triphosphate
PPP	pentose phosphate pathway
TCA	tricarboxylic acid
