# Supplementary material for: Mn3O4 Nanozyme Soaking Improved Wheat Germination and Yield Under Salt Stress
Source: Plants (Basel). 2026 Jul 9;15(14):2124. doi: 10.3390/plants15142124 (PMC13415251; doi:10.3390/plants15142124)
Supplement: Supplementary file 1 [file plants-15-02124-s001.zip › plants-4415041-supplementary.pdf]

Supporting Material

Supplementary Figures

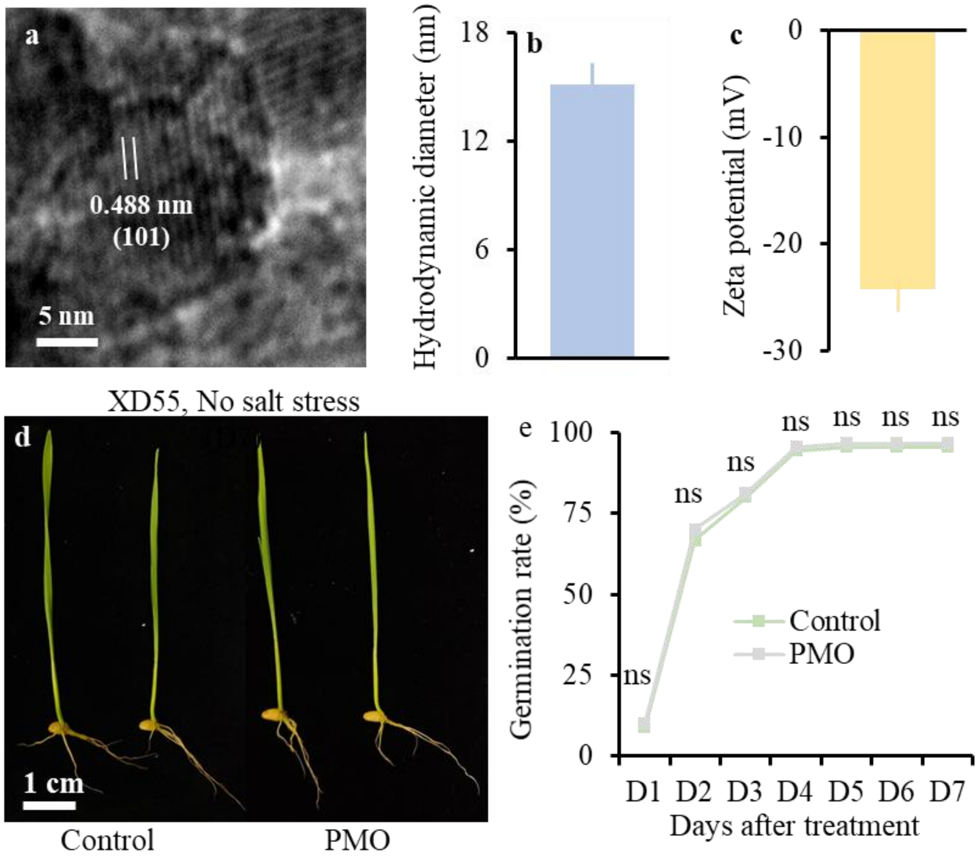

Figure S1. Characteristics of PMOs and phenotypic performance of wheat seeds with and without PMO soaking under no-salt conditions (7 days). (a) TEM image of the PMO lattice. (b) Hydrodynamic diameter of PMOs. (c) Zeta potential of PMOs. (d) Phenotype of wheat seedlings grown from seeds with and without PMO soaking under no-salt conditions (7 days). (e) Germination rate of wheat seedlings grown from seeds with and without PMO soaking under no-salt conditions (7 days). Mean ± SE. ns indicates no significant difference.

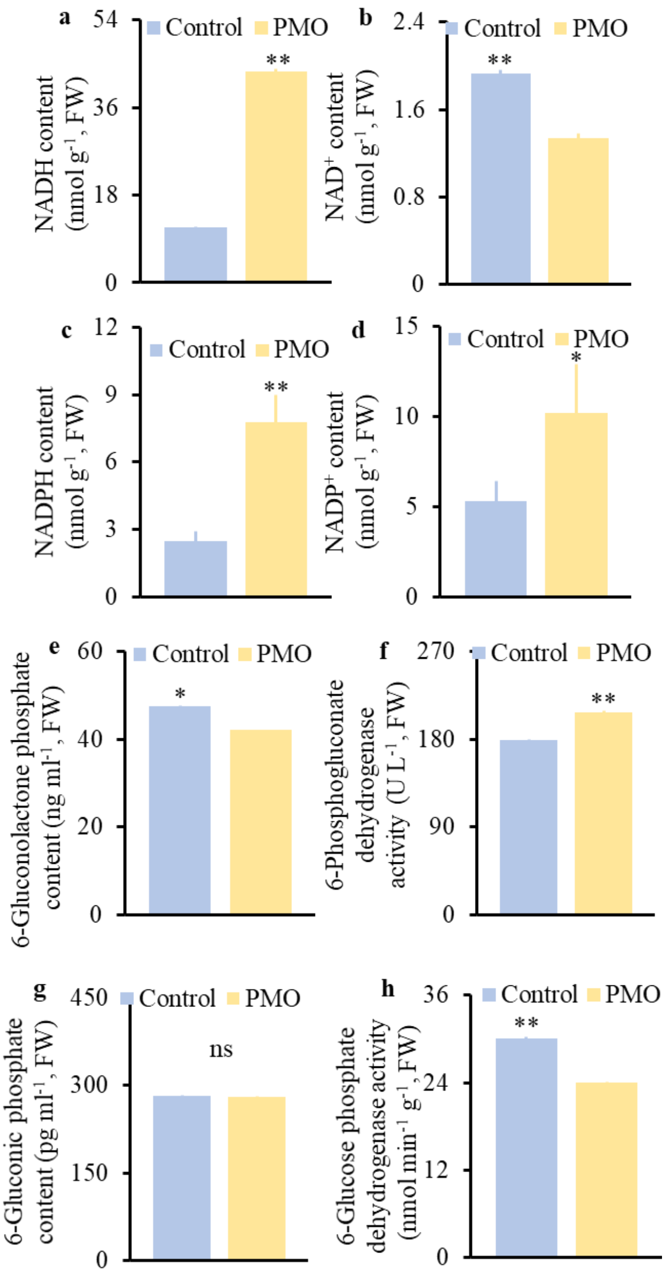

Figure S2. Key metabolite contents and enzyme activities involved in the pentose phosphate pathway in wheat seeds treated with and without PMOS under 300 mM NaCl (7 days): NADH content (a), NAD+ content (b), NADPH content (c), NADP+ content (d), 6-gluconolactone phosphate content (e), 6-phosphogluconate dehydrogenase activity (f), 6-gluconic phosphate content (g), and 6-glucose phosphate dehydrogenase content (h). Mean ± SE (n = 3). * means $P < 0.05$, ** means $P < 0.01$.

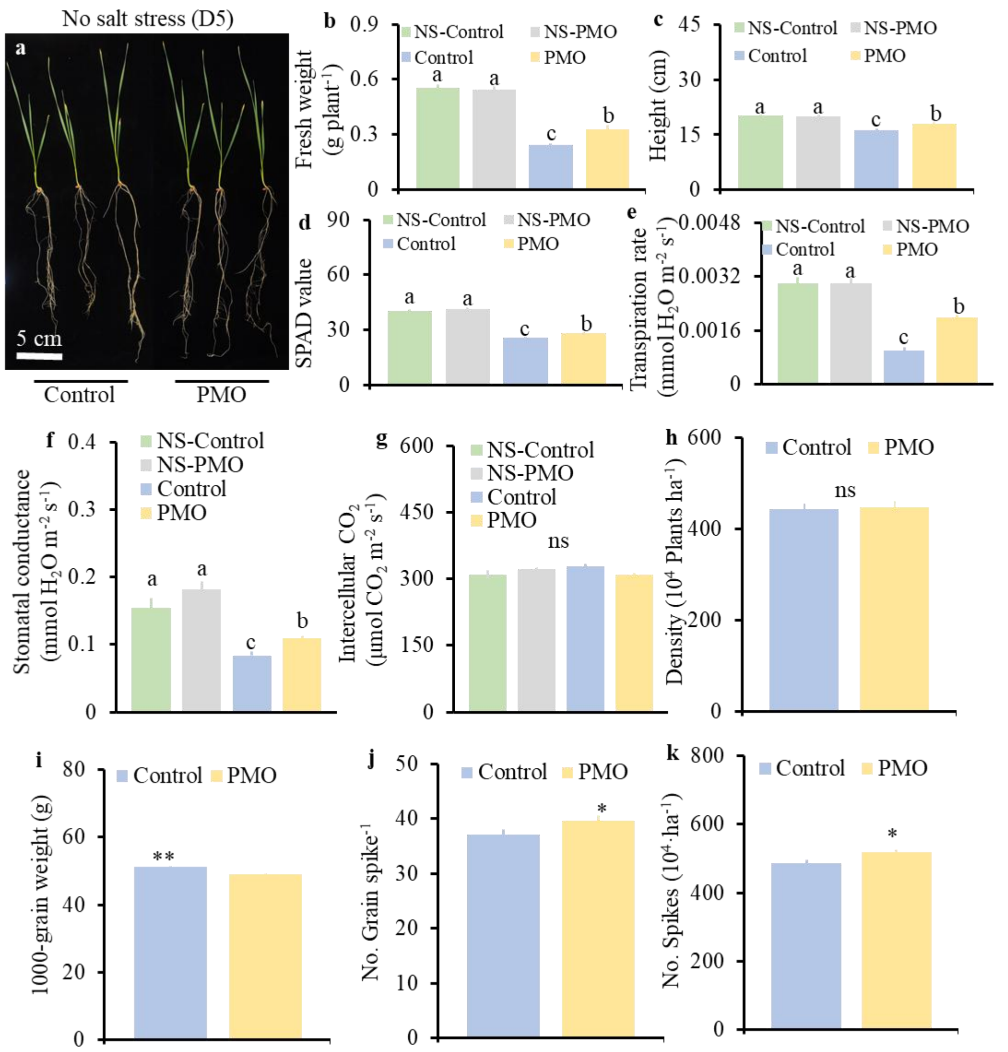

Figure S3. Phenotypic performance and photosynthesis parameters of wheat seedlings grown from seeds with and without PMO soaking. (a) Phenotypic performance of wheat seedlings grown from seeds with and without PMO soaking under no-salt conditions (5 days). (b–d) Fresh weight (b, n = 10), height (c, n = 10), and SPAD value (d, n = 10) of wheat seedlings grown from seeds with and without PMO soaking under no-salt conditions (5 days). (e–g) Transpiration rate (e), stomatal conductance (f), and intercellular CO_2 (g) of wheat seedlings grown from seeds with and without PMO soaking (5 days). (h–k) Density (h), 1000-grain weight (i), number of grains (j), and number of spikes (k) in mature stage wheat grown from seeds with and without PMO soaking under salt stress. Mean ± SE (n = 9). * means $P < 0.05$. Different lowercase letters indicate $P < 0.05$. ns indicates no significant difference.

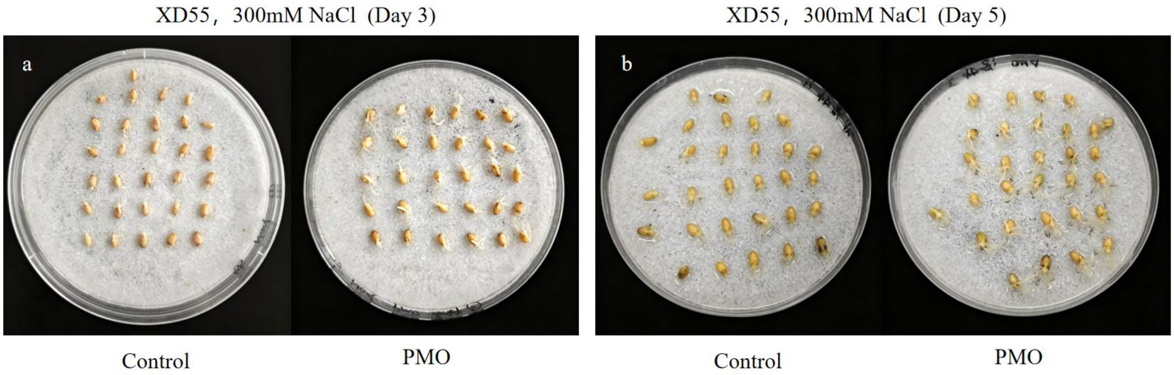

Figure S4. Representative photographs of wheat germination communities in the PMO-treated and control groups, taken on the 3rd and 5th days after soaking.

Materials and methods

Methods for Kit-Based Assays

H_2O_2 content was determined by titanium sulfate colorimetry (415 nm); O_2^- content was determined by hydroxylamine hydrochloride–p-aminobenzenesulfonic acid–α-naphthylamine colorimetry (530 nm); MDA content was determined by thiobarbituric acid (TBA) colorimetry (difference between 532 nm and 600 nm); POD activity was determined by guaiacol colorimetry (470 nm); CAT activity was determined by UV spectrophotometry (240 nm); SOD activity was determined by the WST-8 method (450 nm); soluble protein content was determined by the BCA method (562 nm); proline content was determined by acidic ninhydrin colorimetry (520 nm); APX activity was determined by UV spectrophotometry (290 nm, AsA oxidation rate); GR activity was determined by UV spectrophotometry (340 nm, NADPH decrease rate); DHAR activity was determined by UV spectrophotometry (265 nm, DHA decrease rate); MDHAR activity was determined by UV spectrophotometry (340 nm, NADH decrease rate); MDHA content was determined by TMB colorimetry (450 nm); AsA and DHA contents were determined by bathophenanthroline colorimetry (534 nm); GSH and GSSG contents were determined by the DTNB method (412 nm); HK activity was determined by UV spectrophotometry (340 nm, NADPH generation rate); PFK activity was determined by UV spectrophotometry (340 nm, NADH decrease rate); PGK and GAPDH activities were determined by UV spectrophotometry (340 nm, NADH consumption); PK activity was determined by UV spectrophotometry (340 nm, NADH decrease rate); PEPC activity was determined by UV spectrophotometry (340 nm, NADH decrease rate); the contents of EMP pathway metabolites (6PG, F6P, F-1,6-BP, GAP, 1,3-DPG, 3-PG, 2-PG, and PEP) were determined by competitive ELISA (450 nm); 6-phosphogluconate dehydrogenase and 6-phosphogluconolactonase activities were determined by NADPH colorimetry (450 nm); the contents of PPP pathway metabolites (6-phosphogluconolactone and 6-phosphogluconic acid) were determined by competitive ELISA (450 nm); NAD^+ and NADH contents were determined by alcohol dehydrogenase–WST-8 colorimetry (450 nm); PDH activity was determined by the WST-8 reduction method (450 nm); CS activity was determined by the DTNB method (412 nm); ICDHm activity was determined by UV spectrophotometry (340 nm, NAD^+ reduction rate); MDHm activity was determined by UV spectrophotometry (340 nm, NADH decrease rate); A-CoA and SCoA contents were determined by competitive ELISA (450 nm); the contents of oxaloacetic acid, pyruvic acid, and α-ketoglutaric acid were determined by phenylhydrazine derivatization–high-performance liquid chromatography (324 nm); the contents of malic acid, isocitric acid, citric acid, succinic acid, fumaric acid, and cis-aconitic acid were determined by

high-performance liquid chromatography (214 nm); sucrose content was determined by resorcinol colorimetry (480 nm); starch and glucose contents were determined by anthrone colorimetry (620 nm); and soluble sugar content was determined by anthrone colorimetry (620 nm). For each parameter, quantification was performed using standard curves prepared with kit-provided standards. Reagent blanks were included in each assay run to correct background absorbance, and all samples were measured in three technical replicates to ensure precision. Detailed protocols were followed according to the respective kit instructions (Suzhou Michy Biomedical Technology Co., Ltd., Suzhou, China).
